# Clusterin secreted from astrocyte promotes excitatory synaptic transmission and ameliorates Alzheimer’s disease neuropathology

**DOI:** 10.1186/s13024-021-00426-7

**Published:** 2021-01-31

**Authors:** Fading Chen, Dan B. Swartzlander, Anamitra Ghosh, John D. Fryer, Baiping Wang, Hui Zheng

**Affiliations:** 1 Huffington Center on Aging, Baylor College of Medicine, Houston, TX 77030 USA; 2 Present address: Stanley Center for Psychiatric Research, Broad Institute of MIT and Harvard, Cambridge, MA 02142 USA; 3 Department of Neuroscience, Mayo Clinic, Scottsdale, AZ 85259 USA; 4 Department of Molecular and Human Genetics, Baylor College of Medicine, Houston, TX 77030 USA

**Keywords:** Alzheimer’s disease, Amyloid, Astrocyte, Clusterin, Mice, Synaptic transmission

## Abstract

**Background:**

Genome-wide association studies have established clusterin (CLU) as a genetic modifier for late-onset Alzheimer’s disease (AD). Both protective and risk alleles have been identified which may be associated with its expression levels. However, the physiological function of clusterin in the central nervous system remains largely unknown.

**Methods:**

We examined Clu expression in mouse brains by immunohistochemistry and high-resolution imaging. We performed electrophysiological recordings and morphological analysis of dendritic spines in wild-type and *Clu* knockout mice. We tested synaptic function of astrocytic Clu using neuron-glia co-cultures and by AAV-mediated astroglial Clu expression in vivo. Finally, we investigated the role of astrocytic Clu on synaptic properties and amyloid pathology in 5xFAD transgenic mouse model of AD.

**Results:**

We show that astrocyte secreted Clu co-localizes with presynaptic puncta of excitatory neurons. Loss of Clu led to impaired presynaptic function and reduced spine density in vivo. Neurons co-cultured with Clu-overexpressing astrocytes or treated with conditioned media from HEK293 cells transfected with Clu displayed enhanced excitatory neurotransmission. AAV-mediated astroglial Clu expression promoted excitatory neurotransmission in wild-type mice and rescued synaptic deficits in *Clu* knockout mice. Overexpression of Clu in the astrocytes of 5xFAD mice led to reduced Aβ pathology and fully rescued the synaptic deficits.

**Conclusion:**

We identify Clu as an astrocyte-derived synaptogenic and anti-amyloid factor; the combination of these activities may influence the progression of late-onset AD.

**Supplementary Information:**

The online version contains supplementary material available at 10.1186/s13024-021-00426-7.

## Introduction

Alzheimer’s disease (AD) is a neurodegenerative disease characterized by progressive deterioration of memory and cognitive function, the accumulation of β-amyloid (Aβ) plaques and neurofibrillary tangles and synaptic and neuronal loss leading to dementia. Synapse degeneration is widely accepted as an early and causal event in AD pathogenesis [1]. While genetic studies have discovered rare mutations in genes (*APP*, *PSEN1* and *PSEN2*) that cause familial early-onset AD [2], the vast majority of AD cases are late-onset (LOAD) influenced by a large number of low-penetrant alleles that may either increase or reduce the risk. Clusterin (*CLU*; also known as apolipoprotein J or ApoJ) was identified by two independent genome-wide association studies as one of the most significant LOAD genes [3, 4]. Subsequent studies revealed that the major rs11136000^C^ allele confers an increased risk for AD and is associated with reduced *CLU* expression whereas the minor rs11136000^T^ allele is associated with increased *CLU* expression and reduced AD risk [5–8]. The C allele is also associated with faster cognitive decline in presymptomatic stages of AD [9], poorer memory scores in both AD and non-demented elderly [10], as well as altered brain structure and circuit activity in young healthy adults [11, 12], suggesting that changes of neural network early in life may affect cognitive function and AD progression at later stages in C allele carrying individuals. A better understanding of CLU’s function, particularly in synaptic regulation, may thus provide mechanistic insight into its genetic association in LOAD.

CLU is a multifunctional protein that can be present intracellularly or secreted as lipoprotein particles [13]. In the peripheral system, CLU is known to participate in a variety of cellular processes, ranging from extracellular misfolded protein clearance [14], lipid transport [13], complement inhibition [15], to oxidative stress and cell death [16]. CLU is highly expressed in the brain but its physiological function in the central nervous system is poorly understood. Elevated levels of CLU have been observed in brain tissues and cerebrospinal fluid (CSF) of AD patients [17–20]. However, the molecular underpinning and functional consequences of this increase is not clear. It is known that CLU is present in the Aβ plaques and in vessels of cerebral amyloid angiopathy (CAA) [21–23]. As an extracellular chaperone, CLU has been shown to bind to Aβ and is involved in Aβ deposition, fibrillogenesis, and clearance [24, 25]. In vivo studies using APP transgenic mice crossed with *Clu* knockout mice revealed that loss of Clu leads to reduced dense core plaques and heightened CAA [26, 27], demonstrating an important role of Clu in modulating both Aβ deposition in the brain and its clearance through the blood brain barrier. Nevertheless, since CLU is upregulated in AD conditions, how CLU overexpression affects AD pathophysiology in vivo is a critical question remains to be addressed.

Here we utilized *Clu* knockout mice and AAV-mediated gene delivery to drive the expression of Clu in astrocytes of WT and 5xFAD mice and investigated the role of Clu in synaptic transmission and amyloid pathology. We demonstrate that Clu secreted from astrocyte is required for proper excitatory synaptic transmission and spine density under physiological conditions and attenuates amyloid pathology and associated neuronal toxicity in an AD mouse model.

## Methods

### Mice and AAV delivery

The Aldh1L1-GFP [28], *Clu* knockout [29], Thy1-EGFP [30], and 5XFAD mice [31] have been described previously. The mice were obtained from the Jackson Laboratory (Bar Harbor, ME, USA) and have been backcrossed to C57BL/6 background for at least ten generations. Both female and male mice were used unless otherwise specified. For AAV-mediated gene delivery, P3 pups were anesthetized via hypothermia and injected free-hand into the bilateral ventricles with 5 × 10^10^ viral particles per side of AAV2/8-GFAP-GFP or AAV2/8-GFAP-Clu using a 28-gauge needle attached to a Hamilton syringe.

All experiments were performed in accordance with procedures approved by the Institutional Animal Care and Use Committee of Baylor College of Medicine and ARRIVE guidelines.

### Clu conditioned medium (CM) preparation

Clu CM was collected from cultured HEK293 cells. Briefly, ~ 90% confluent HEK293 cells were transfected with plasmid containing mouse Clu cDNA or empty vector (Origene) using Lipofectamine 3000 (Invitrogen) following the manufacturer’s instructions. Two days after transfection, the cells were washed with warm DPBS and placed in minimum medium containing phenol red-free Neurobasal, glutamax and Pen/Strep for 3 days. The medium was collected, concentrated 30–50 folds using centrifugal filter (Millipore, MWCO 10 K), and applied to primary cultured neurons at 30–40 μg/ml.

### Primary neuronal culture

Dissociated cortical neurons were harvested from P0 mice. Briefly, newborn pups were decapitated and dissected in ice-cold dissection medium (HBSS with 10 mM HEPES, 0.6% glucose, 1% v/v Pen/Strep). Dissected mouse cortex was cut into small pieces, followed by enzymatic digestion using papain dissociation system (Worthington Biochemical Corporation) according to manufacturer’s instructions. Dissociated neurons were cultured on glass coverslips coated with poly-D-lysine (PDL) and laminin at a density of 200,000 cells/cm^2^. Neuronal culture media consisted of Neurobasal medium supplemented with 1x Glutamax, 1000 μ/ml Pen/Strep, 1x B27 supplement. Cultures were maintained at 37 °C in 5% ambient CO_2_. Half medium was changed every other day.

### Primary astrocyte culture

Mouse cortex was isolated from P3 pups for astrocyte culture. Briefly, the brain was dissected in ice-cold dissection medium (HBSS with 10 mM HEPES, 0.6% glucose, 1% v/v Pen/Strep) and finely minced. The tissue was digested in 2.5% trypsin at 37 °C for 15 min, followed by the addition of trypsin inhibitor (1 mg/ml) and DNase. The tissue was then centrifuged at 1000 g for 5 min, triturated, re-suspended in astrocyte culture medium (DMEM + 10% FBS + 1000 μ/ml Pen/Strep). Cells were plated on PDL coated T75 flasks at 50,000 cells/cm^2^. After the culture reached confluency, the flasks were rocked at 250 rpm overnight at 37 °C to remove unwanted cell types (microglia, oligodendrocytes, neurons, and fibroblasts). After recovery in fresh medium for 24 h, astrocytes were trypsinized in 0.25% trypsin/EDTA and re-plated on PDL coated glass coverslip for future experiments.

### Primary astrocyte AAV transduction and neuron-astrocyte co-culture

For AAV transduction in primary astrocytes, the viral particles were added to the reduced serum medium (DMEM + 2% FBS) at 500,000 multiplicity of infection in half of the usual volume. Following 4–6 h of incubation, equal amount of fresh astrocyte medium (DMEM + 10% FBS) was added to the culture. The medium was changed 2 days later. Astrocytes were cultured for an additional 2 weeks to reach optimal Clu or GFP expression. Transduction efficiency was observed at > 90%.

Primary cortical neurons harvested from P0 pups were seeded on top of transduced astrocyte monolayer to form neuron-astrocyte co-culture. The co-culture was maintained in the neuronal culture media. Half of the medium was replaced with fresh medium every other day.

### Immunoblotting

For Western blot, cells and brain tissues were lysed in RIPA buffer (TBS with 1% NP-40, 1% sodium deoxycholic acid, 0.1% sodium dodecyl sulfate, and protease/phosphatase inhibitor cocktails). Lysates were sonicated (six pulses at 50% duty cycle) and incubated on ice for 30 min. Samples were then centrifuged at 20,000 g for 20 min. Supernatants were collected and quantified using a Pierce BCA Protein Assay kit (Thermo Fisher). 20-μg protein samples were loaded onto 12% SDS-PAGE gels and then transferred to nitrocellulose membranes (Bio-Rad). Membranes were blocked in 5% nonfat milk in PBST (PBS + 0.1% Tween 20). Blots were probed with primary antibodies overnight at 4 °C. After washing with PBST, blots were then probed with a donkey anti-goat IgG (H + L) conjugated to IRDye 800CW (LI-COR) secondary antibody. The signals were detected on the LI-COR Odyssey (LI-COR, Bad Homburg, Germany).

### Immunofluorescence staining

Animals were perfused transcardially with 4% paraformaldehyde (PFA) in 0.1 M phosphate-buffered saline (PBS), pH 7.4, under ketamine (300 mg/kg) and xylazine (30 mg/kg) anesthesia. Brains were harvested, post-fixed in the same fixative overnight at 4 °C, dehydrated with 30% sucrose in PBS, and serially sectioned at 40 μm on a sliding microtome (Leica). For immunofluorescence, sections were permeabilized in PBS/0.1% Triton X-100 for 10 min and blocked with 4% normal goat serum in PBS/0.1% Triton X-100 for 1 h at room temperature. Sections were then incubated with primary antibodies in 2% serum in PBS/0.1% Triton X-100 overnight at 4 °C: GFAP (1:1000, EMD Millipore); Iba-1 (1:800, Wako); Clu (1:1000, R&D systems); vGlut1 (1:1000, AbCam); GAD67 (1:1000, Millipore); PSD-95 (1:1000, BD Transduction Laboratories); 6E10 (1:1000, BioLegend); CD68 (1:500, BioLegend). Sections were then washed and incubated with Alexa Fluor 594- or Alexa Fluor 647-conjugated secondary antibodies (Invitrogen) for 1 h at room temperature. After washing with PBS, sections were incubated with either DAPI to stain the nucleus or methoxy-X04 (Tocris) to stain the Aβ plaques. Images were captured using a Laser-Scanning Confocal Microscopy (Leica).

### Quantitative analyses of dendritic spines

Four-month-old Thy1-GFP mice on wild-type or *Clu* KO background were perfused. Brain slides were individually coded and randomly ordered for image acquisition. High-resolution images of tertiary and quaternary branches of apical dendrites of pyramidal neurons (3–5 randomly selected neurons per mouse/4 mice per group), displaying GFP fluorescence throughout the entire dendritic tree, were acquired using a Leica laser-scanning confocal microscopes with oil immersion at 63× (NA 1.4) objective and 5× digital zoom. Series of optical sections were acquired in the z-axis at 0.08 μm intervals through individual apical dendritic branches. The dendritic fragments and spines were traced and the classification and quantification of spine types were performed by the 3-D IMARIS software. Spine density was calculated by quantifying the number of spines per 10 μm of dendritic segment.

### Transcriptome and gene ontology analysis

The NanoString nCounter Analysis system was used to analyze the total RNA extracted from neuronal cultures. Normalization and calculation of differential expression was performed using nSolver 4.0 with default settings (NanoString Platform, NanoString Technologies). Gene clusters were analyzed using protein analysis through evolutionary relationships (PANTHER) Overrepresentation Test based on the gene ontology (GO) database (http://www.pantherdb.org/). Reactome pathway analysis (https://reactome.org/) was used to determine the enriched pathways of top expressed genes.

### Electrophysiology

Briefly, mice were anesthetized and perfused with ice-cold sucrose-based cutting solution (250 mM Sucrose, 2.5 mM KCl, 1.25 mM NaH_2_PO_4_, 25 mM NaHCO_3_, 10 mM MgCl_2_, 1 mM CaCl_2_, and 10 mM glucose, saturated with 95% O_2_ and 5% CO_2_). 350 μm horizontal hippocampus sections were cut using a Leica VT1200S vibratome and slices were allowed to recover for at least 1 h in recording artificial cerebrospinal fluid (ACSF) (125 mM NaCl, 2.5 mM KCl, 1.25 mM NaH_2_PO_4_, 25 mM NaHCO_3_, 1 mM MgCl_2_, 2 mM CaCl_2_, and 10 mM glucose) bubbled with 95%O_2_/5%CO_2_ at all times to maintain consistent oxygenation and pH. To record mEPSCs and mIPSC, pipettes were filled with a cesium-substituted intracellular solution (mM): 10 CsCl, 105 CsMeSO3, 0.5 ATP, 0.3 GTP, 10 Hepes, 5 glucose, 2 MgCl2 and 1 EGTA. For mEPSC recording, neurons were held at − 70 mV in the present of 0.5 μM TTX and 50 μM Picrotoxin. For mIPSC recording, neurons were held at 10 mV in the presence of 0.5 μM TTX, 20 μM DNQX and 50 μM APV. Whole cell patch clamp recordings were acquired (MultiClamp 700B, Axon Instruments), digitized at 20 kHZ (Digidata 1440A, Axon Instruments), filtered at 2 kHz, and acquired with pClamp software (Axon Instruments). Pipette resistance ranged from 4 to 7 MΩ. Access resistance ranged from 8 to 20 MΩ. Recordings were discarded if the initial leak current was above 200 pA or changed by more than 20% over the course of recording. Offline analysis was performed using Clampfit (Axon Instruments).

To record field excitatory postsynaptic potentials (fEPSPs), stimulation of Schaffer Collaterals from the CA3 region was performed with bipolar electrodes, while borosilicate glass capillary pipettes filled with recording ACSF (2–3.5 MΩ resistance) were used to record fEPSPs in the CA1 region. Signals were amplified using a MultiClamp 700B amplifier (Axon Instruments), digitized using a Digidata 1440A (Axon Instruments) with a 2 kHz low pass filter and a 3 Hz high pass filter and then captured and stored using Clampex 10.4 software (Axon Instruments) for offline data analysis using Clampfit (Axon Instruments).

### Statistical analysis

All data were analyzed with GraphPad Prism v.7 and are presented as Mean ± SEM (*p < 0.05, **p < 0.01, and ***p < 0.001). Power analysis was performed using a confidence interval of α = 0.05. Pairwise comparisons were analyzed using two-tailed Student’s t test. For multiple comparisons in fEPSP recording, ANOVA followed by Tukey’s post hoc testing was used.

## Results

### Clu is expressed in astrocytes and also targeted to the presynaptic sites

**Fig. 1:**
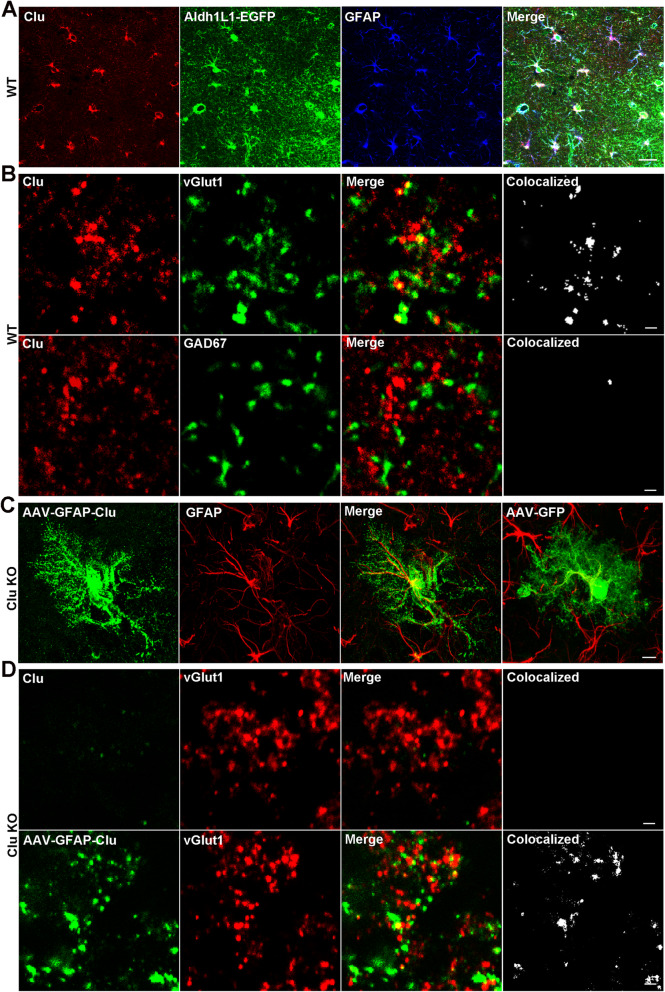
Clu is expressed in astrocyte and targeted to the excitatory synaptic sites. **a** Immunofluorescence staining of hippocampal area CA1 of a Aldh1L1-EGFP mouse showing co-localization of endogenous Clu with EGFP-marked and GFAP-positive astrocytes. **b** Higher magnification confocal images showing abundant co-localization of Clu with excitatory synaptic structures marked by vGlut1 but not with inhibitory synapses marked by GAD67. **c** Immunofluorescence staining showing astroglial expression of Clu or GFP in *Clu* knockout mice 3 weeks after AAV-GFAP-Clu or AAV-GFAP-GFP infection. **d** Co-immunofluorescence staining of Clu and vGlut1 and high resolution confocal imaging of *Clu* KO mice infected with AAV-GFAP-Clu, showing co-localization of Clu puncta with vGlut1. Non-infected *Clu* KO (Clu) mice were used as controls. Scale bars, 20 μm (**a**), 5 μm (**c**) and 1 μm (**b**) & (**d**)

We first examined the expression pattern of endogenous Clu using the Aldh1L1-GFP reporter mice [28]. Consistent with the previous reports [32, 33], immunostaining using an anti-Clu antibody showed that Clu is highly expressed in GFP-marked astrocytes that are also GFAP positive (Fig. 1a). In addition, high resolution confocal imaging detected Clu immunoreactivity as discrete synapse-like puncta outside astrocytes (Fig. 1b). Co-immunostaining with antibodies against the vesicular glutamate transporter (vGlut1) or GAD67, which marks presynaptic terminals of excitatory and inhibitory neurons, respectively, documented that 17.75% vGlut1-positive puncta contained Clu, whereas there was minimum co-localization between GAD67 and Clu (Fig. 1b). This result suggests that a fraction of Clu is targeted to the synaptic site where it is preferentially associated with glutamatergic synapses.

To distinguish whether the synaptic Clu puncta are secreted from astrocytes or they are neuronal derived, we delivered low dose AAV-GFAP-Clu virus to the lateral ventricles of *Clu* knockout (KO) mouse brains at postnatal day 3 (P3) to sparsely label astrocytes [34], and examined the expression pattern of the astroglial expressed AAV-Clu in the absence of neuronal Clu 28 days post infection. Co-immunostaining of Clu with GFAP observed prominent Clu expression in GFAP-positive astrocytes (Fig. 1c). Noticeably, Clu immunoreactivity filled the entire astrocyte territory in *Clu* KO mouse, similar to the territory covered by GFP signals in AAV-GFAP-GFP infected astrocyte (Fig. 1c). However, in contrast to the smooth GFP staining pattern, Clu immunostaining revealed discrete punctate pattern both inside and outside of astrocyte territory, demonstrating that these puncta are produced and secreted from infected astrocytes. Co-immunostaining of Clu with vGlut1 showed that, similar to endogenous Clu, Clu secreted from infected astrocytes also co-localized with vGlut1 at the synaptic sites (Fig. 1d). Taking together, these data demonstrate that Clu secreted from astrocyte target to the presynaptic sites of excitatory neurons.

### *Clu* deficiency alters excitatory synaptic transmission and spine density in vivo

**Fig. 2:**
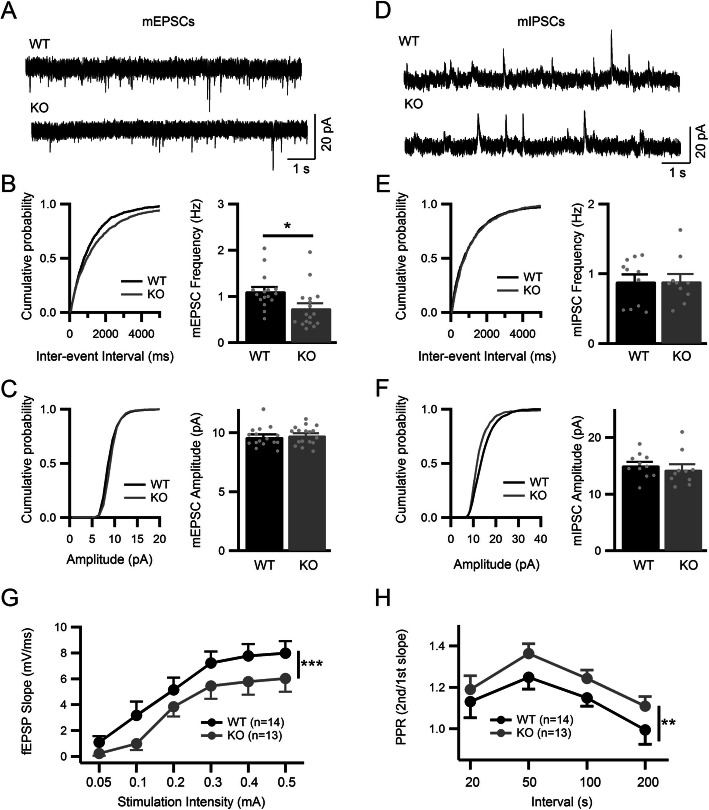
Impaired excitatory neurotransmission in the hippocampus of *Clu* null mice. **a** mEPSC sample traces from WT and *Clu* KO neurons voltage clamped at − 65 mV (scale bar, 20pA/2 s). **b** Cumulative distribution of inter-event interval from all mEPSCs events of 16 WT and 17 KO neurons (left) and average mEPSC frequency (right). **c** Cumulative distribution of amplitude of all neurons (left) and average mEPSC amplitude (*n* = 16 for WT and *n* = 17 for KO). **d** mIPSC sample traces from WT and KO neurons voltage clamped at 10 mV (scale bar, 20pA/2 s). **e** Cumulative distributions of inter-event interval from all mIPSCs events of 11 WT and 10 KO neurons (left) and average mIPSC frequency (right). **f** Cumulative distribution of amplitude of all neurons (left) and average mEPSC amplitude (*n* = 11 for WT and *n* = 10 for KO). **g** Relationship between fEPSP slope and stimulating current intensity in WT and KO hippocampi showing a significant decrease in CA3-CA1 excitatory transmission (*n* = 14 for both WT and KO). **h** Average paired pulse ratio (PPR) of fEPSP slope in CA3-CA1 synapse showing increased PPR in KO compared to WT (*n* = 17 for WT, *n* = 13 for KO). All data are presented as mean ± SEM. Average number of animals tested: *N* = 3 per group for whole cell recording and *N* = 4 per group for field recording.*p < 0.05; ∗∗*p* < 0.01; ∗∗∗*p* < 0.001. Student’s t-test (**b**), (**c**), (**e**), (**f**); Two-way ANOVA (**g**) & (**h**)

The synaptic targeting supports a functional role of astrocytic Clu in excitatory synaptic transmission. To provide direct support, we performed ex vivo recordings of miniature excitatory and inhibitory postsynaptic currents (mEPSCs and mIPSCs) from hippocampal granule neurons of P28–30 of *Clu* KO mice and their wild-type (WT) littermate controls. We observed a significant decrease of mEPSC frequency in *Clu* KO neurons compared to WT controls as indicated by a right shift in cumulative probability of inter-event interval and a reduction in average frequency (Fig. 2a & b), while both the cumulative probability and average mEPSC amplitude were comparable between WT and *Clu* KO neurons (Fig. 2c). Neither the mIPSC frequency nor amplitude were altered in *Clu* KO neurons compared to WT controls (Fig. 2d-f), suggesting that astrocytic Clu selectively regulates excitatory but not inhibitory synaptic transmission. The specific effect on mEPSC frequency but not amplitude indicates that Clu does not affect the postsynaptic response but rather acts by regulating presynaptic release probability and/or synapse density.

To probe the mechanisms further, we examined pathway-specific effect by recording field excitatory postsynaptic potentials (fEPSPs) of the Schaffer Collateral pathway of acute hippocampal slices. Compared with littermate WT controls, the slope of fEPSP plotted as a function of stimulus intensity was significantly lower in *Clu* KO brain slices (Fig. 2g), indicating reduced total synaptic strength. To determine if this is due to alterations in presynaptic neurotransmitter release, we measured paired-pulse ratio (PPR), which is inversely related to probability of transmitter release, by recording fEPSPs evoked by two stimuli with various intervals. We detected significantly larger PPR in *Clu* KO brain slices compared to WT controls (Fig. 2h), indicating that the probability of transmitter release is lower in *Clu* KO hippocampus. These results suggest that Clu regulates excitatory synaptic transmission, at least in part, by modulating presynaptic release probability.

**Fig. 3:**
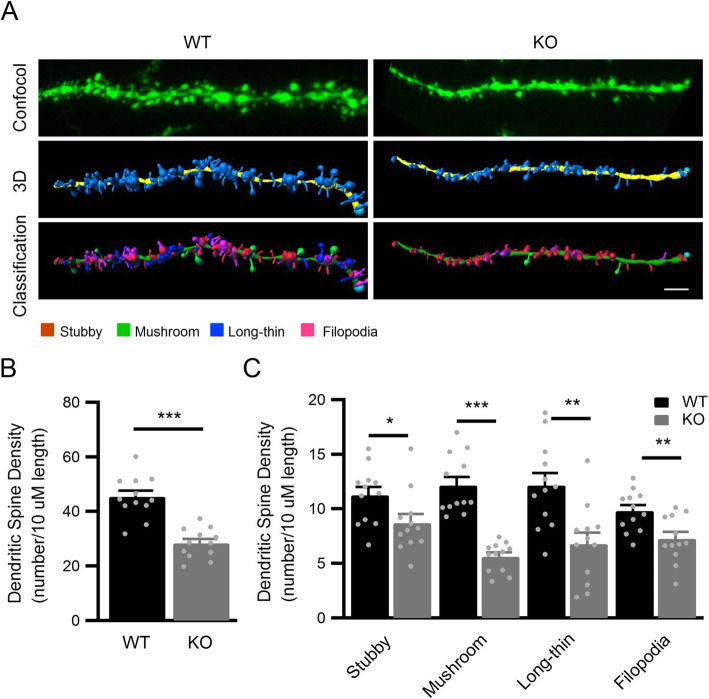
Reduced dendritic spine density in hippocampal neurons of *Clu* KO mice. **a** Representative dendritic spines from Thy1-GFP-labeled dendrites in area CA1 of hippocampal pyramidal neurons of four-month-old littermate WT and *Clu* KO mouse brains. IMARIS was used to reconstruct the 3D rendering of the spines on dendrites based on confocal images of GFP-positive neurons and to classify the four spine types (mushroom, stubby, long-thin, and filopodia). Scale bar, 2 μm. **b** Total spine density in WT and *Clu* KO mouse brains. **c** Density of each of the four spine types on secondary apical dendrites of WT and *Clu* KO mouse brains. Dendritic segments were derived from 3 random neurons per mouse/ 4 animals for each group. All data are presented as mean ± SEM. *p < 0.05; ∗∗*p* < 0.01; ∗∗∗*p* < 0.001 (Student’s t test)

Since dendritic spines are the major sites of excitatory synaptic transmission, we analyzed spine density in *Clu* KO mice to determine whether there was an anatomical correlate to the reduced mEPSC frequency. We crossed the WT and *Clu* KO mice with a Thy1-GFP reporter [30], and analyzed the number of dendritic spines in sparsely labeled CA1 pyramidal neurons at 4 months of age. In addition, we performed 3D reconstruction of dendritic segments, followed by unbiased classification and quantification of spine types (Fig. 3a). We found significantly reduced total spine density in apical dendrites of *Clu* KO mice compared to WT controls (Fig. 3b). This was attributed by reductions of all spine types including stubby, mushroom, long-thin, and filopodia spines (Fig. 3c). The results combined demonstrate a physiological function of Clu in regulating excitatory synaptic transmission and spine density in vivo.

### Secreted Clu enhances excitatory neurotransmission in vitro

**Fig. 4:**
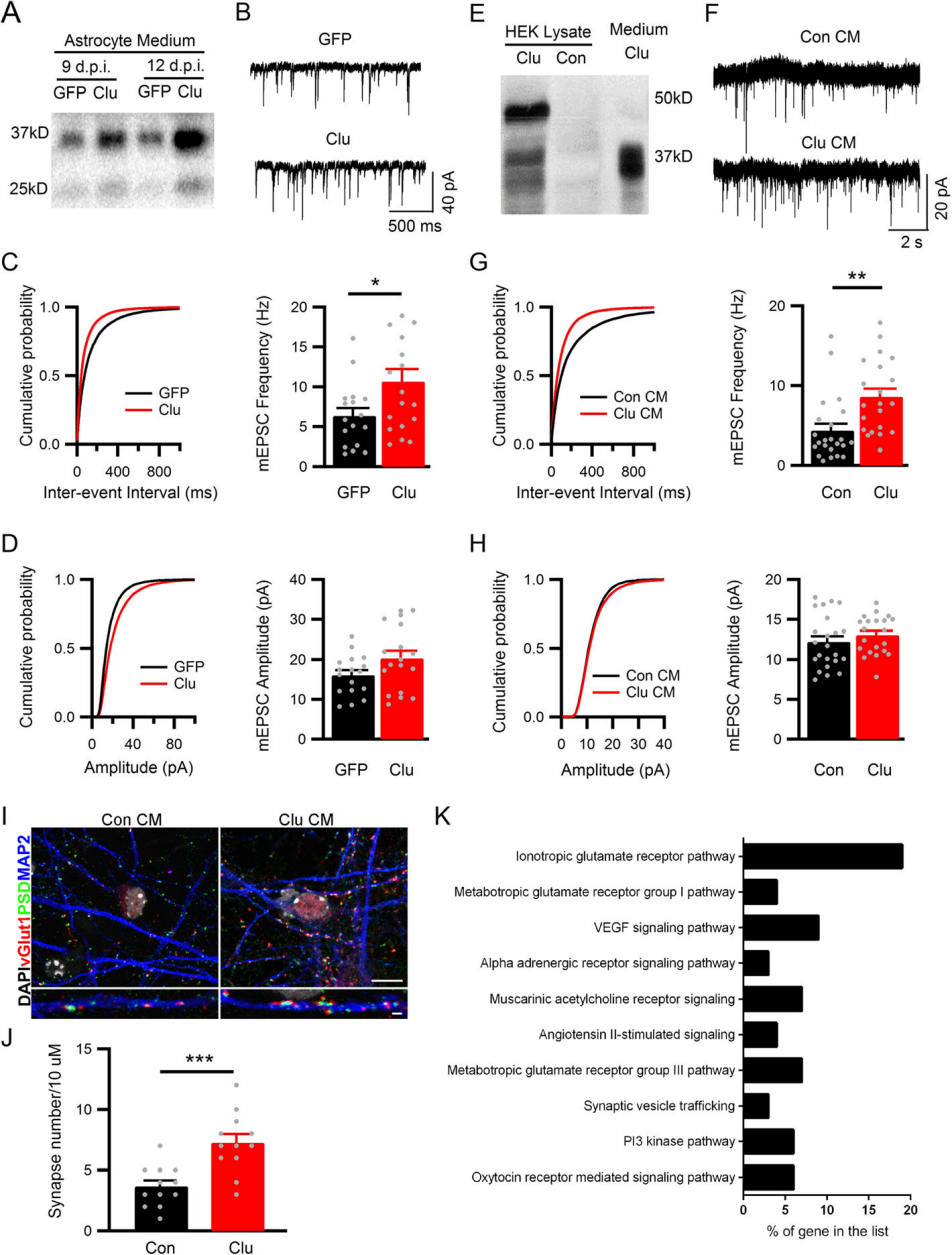
Secreted Clu promotes excitatory synaptic function in vitro. **a** Western blotting of Clu in conditioned medium of primary astrocyte infected with AAV-GFAP-GFP (GFP) or AAV-GFAP-Clu (Clu) at 9 or 12 days post infection (d.p.i.). **b** mEPSC sample traces from primary neurons co-cultured with primary astrocytes infected with AAV-GFAP-GFP or AAV-GFAP-Clu at 12–13 DIV (scale bar, 40 pA/500 ms). **c** Cumulative distribution of inter-event interval from all mEPSCs events of 17 neurons co-cultured with GFP astrocytes and 18 neurons co-cultured with Clu astrocytes (left) and average mEPSC frequency (right). **d** Cumulative distribution of amplitude (left) and average amplitude (right) in neurons co-cultured with GFP (*n* = 17) or Clu (*n* = 18) astrocytes. **e** Western blot analysis of Clu in lysate of HEK293 cells transfected with Clu or vector control (Con) plasmid and in conditioned medium of Clu-transfect HEK293 cells. **f** mEPSC sample traces from primary cultured neurons treated with control conditioned medium (Con CM) or Clu CM at 14–16 DIV (scale bar, 20 pA/2 s). **g** Cumulative distribution of inter-event interval from all mEPSCs events of 23 neurons treated with Con CM and 21 neurons treated with Clu CM (left) and average mEPSC frequency (right). **h** Cumulative distribution of amplitude (left) and average amplitude (right) in neurons treated with Con CM (*n* = 23) or Clu CM (*n* = 21). **i** Representative Confocal images of cultured neurons triple-stained with anti-vGlut1, -PSD95 and -MAP2 antibodies and counterstained with DAPI. Lower panel is a zoomed-in view of a dendritic segment. Colocalized pre- (vGluT1) and post-synaptic (PSD95) puncta reveal sites of excitatory synapses. Scale bar, 10 μm (top), 1 μm (bottom). **j** quantification of synaptic density. **k** Gene Ontology analysis of upregulated genes in Clu vs. control CM treated neuronal cultures. All data are presented as mean ± SEM. *p < 0.05; ∗∗*p* < 0.01; ∗∗∗*p* < 0.001 (Student’s t test)

We next employed in vitro systems to test whether Clu secreted from astrocytes can directly modulate synaptic transmission. We infected primary astrocyte cultures with either AAV-GFAP-GFP or AAV-GFAP-Clu and co-cultured them with primary wild-type neurons. Western blot analysis of astrocyte conditioned media showed increased levels of mature, proteolytically cleaved, Clu proteins in AAV-GFAP-Clu infected cultures compared to GFP infected controls (Fig. 4a). After 12–14 days of co-culture, we performed whole-cell voltage path clamping to record mEPSCs from cultured neurons. Primary neurons co-cultured with AAV-GFAP-Clu infected astrocytes displayed a significant increase of mEPSC frequency but normal amplitude compared with neurons co-cultured with astrocytes infected with control AAV-GFAP-GFP virus (Fig. 4b-d).

To provide further evidence that secreted Clu modulates synaptic transmission, we overexpressed Clu in HEK293 cells and collected conditioned medium. Western blot analysis showed that both the full-length (> 50 KDa) and cleaved bands can be detected in Clu transfected cell lysate, but secreted Clu is almost exclusively present as mature cleaved form (Fig. 4e). We applied the control or Clu expressing conditioned medium to cultured primary neurons at 8 days in vitro (DIV8), and performed whole cell voltage patch clamping to record mEPSCs at DIV12. Consistent with the co-culture data, neurons treated with Clu-expressing conditioned medium displayed a significantly increased mEPSC frequency but normal amplitude (Fig. 4f-h). Together these data demonstrate that secreted Clu positively regulates excitatory synaptic transmission.

Next we asked whether the increased mEPSC frequency is associated with higher synaptic density. We performed co-immunostaining of neurons treated with control or Clu medium with anti-vGlut1 and anti-PSD95 antibodies and quantified the number of co-localized puncta on MAP2-marked dendrites (Fig. 4i). Neurons treated with Clu-expressing conditioned medium displayed more synapses compared to the controls (Fig. 4j), indicating that secreted Clu promotes excitatory synapse formation.

To provide molecular basis for the Clu-dependent effect, we performed gene expression profiling of primary neurons treated with control or Clu-expressing conditioned medium using the NanoString platform. We chose a predesigned neuropathology panel (770 genes) that contained the main neurotransmission and neuron-glia interaction related pathways and detected 68 genes that were differentially expressed by Clu conditioned medium (Supplemental Table 1). Gene Ontology annotations identified the ionotropic metabotropic glutamate receptor pathway genes as the most overrepresented differentially expressed genes (Fig. 4k). To further explore the potential functional implications of these genes, we conducted pathway enrichment analysis based on the Reactome pathway database and found that pathways associated with synaptic transmission, axon guidance and protein-protein interactions at synapses were noticeably enriched (Table S1). All together, these results provide molecular, cellular and functional support that secreted Clu promotes synapse formation and glutamatergic synaptic function in vitro.

### Astrocytic Clu promotes excitatory neurotransmission in vivo and rescues synaptic deficits in *Clu* KO mice

**Fig. 5:**
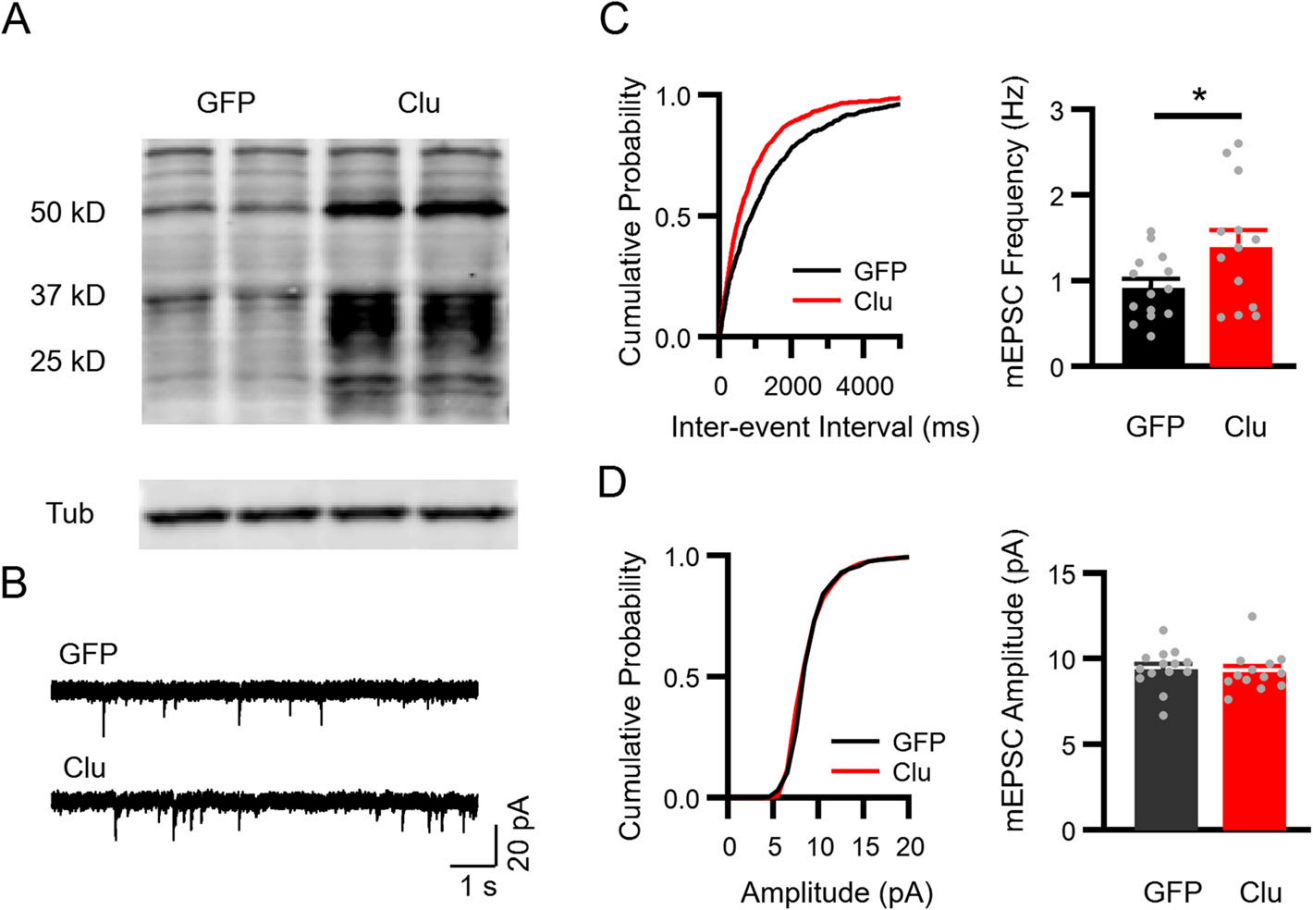
Astrocytic Clu overexpression promotes excitatory synaptic transmission in vivo. **a** Western blotting of Clu expression in hippocampal tissues of wild-type mice injected with AAV-GFAP-GFP or AAV-GFAP-Clu. Tubulin (Tub) was used as a loading control. **b** mEPSC sample traces from hippocampal CA1 pyramidal neurons of GFP or Clu transduced mice (scale bar, 20 pA/1 s). **c** Cumulative distribution of inter-event interval from all mEPSCs events of 14 neurons in mice injected with AAV-GFAP-GFP and 13 neurons injected with AAV-GFAP-Clu (left) and average mEPSC frequency (right). **d** Cumulative distribution of amplitude (left) and average mEPSC amplitude (right) of neurons infected with GFP (*n* = 14) or Clu (*n* = 13) viruses. Number of animals tested: *N* = 3 per group. Data presented are mean ± SEM. *p < 0.05 (Student’s t test)

We proceeded to determine the role of astrocytic Clu in vivo by intraventricular injections of AAV-GFAP-Clu to P3 wild-type mouse brains [35]. AAV-GFAP-GFP injected mice were used as controls, which confirmed widespread and astrocyte-specific expressions in the brain (Supplementary Fig. S1). Western blot analysis showed significant increases of both full-length and cleaved Clu in AAV-GFAP-Clu injected mice (Fig. 5a). We harvested acute brain slices 4 weeks post injection and measured mEPSCs from hippocampal CA1 neurons. Consistent with our in vitro data, we observed that Clu overexpression in astrocytes significantly increased mEPSC frequency, but not amplitude (Fig. 5b-d), demonstrating that astrocyte derived Clu is sufficient to enhance excitatory neurotransmission in vivo.

**Fig. 6:**
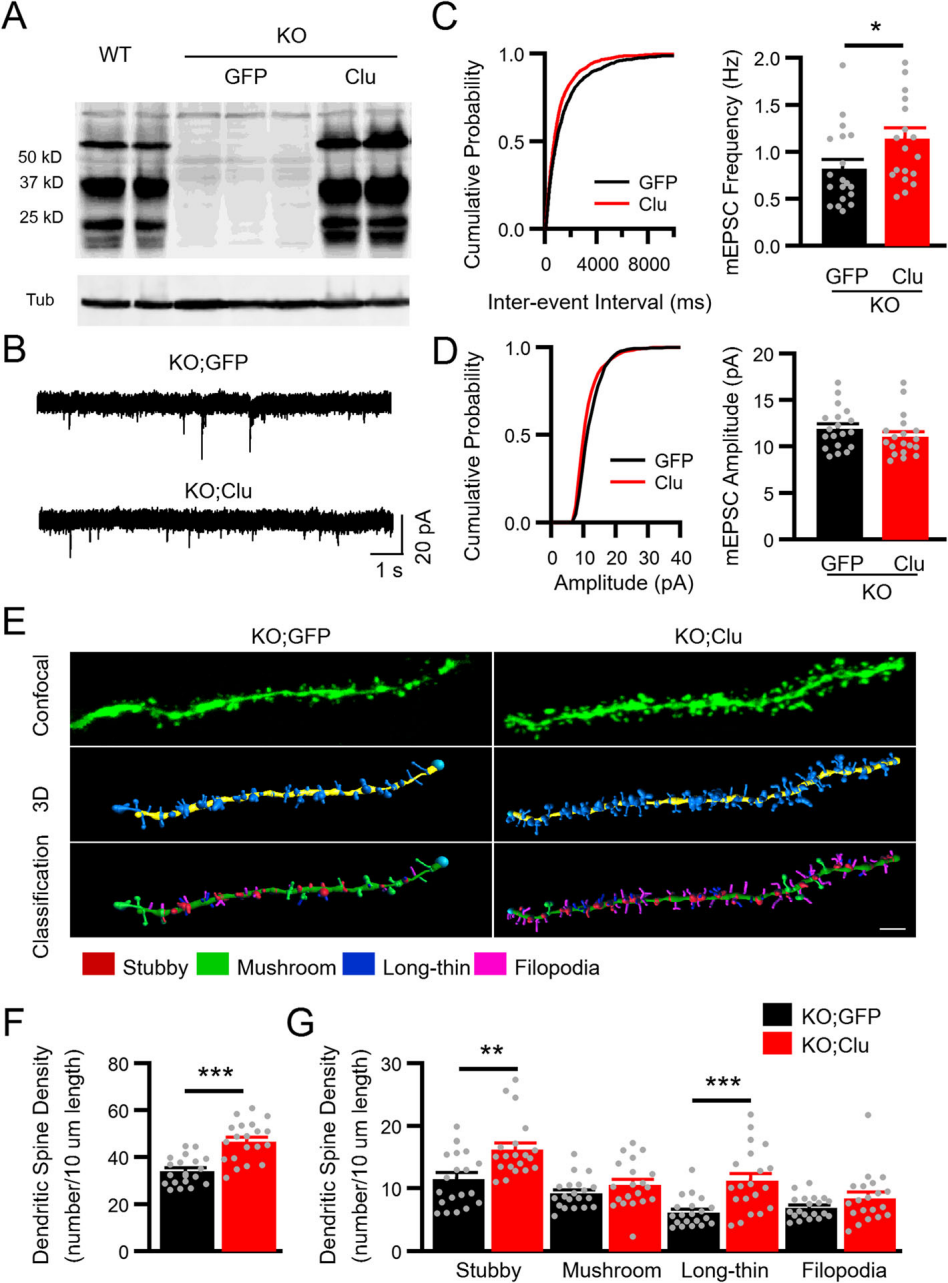
Astrocytic expression of Clu rescues synaptic deficit in *Clu* KO mice. **a** Western blotting of Clu expression in hippocampal tissues of WT and *Clu* KO mice injected with AAV-GFAP-GFP or AAV-GFAP-Clu. Tub: Tubulin loading control. **b** mEPSC sample traces from hippocampal CA1 pyramidal neurons of KO mice injected with AAV-GFAP-GFP (KO;GFP) or AAV-GFAP-Clu (KO;Clu). Scale bar, 20 pA/1 s. **c** Cumulative distribution of inter-event interval (left) from all mEPSCs events of 19 neurons in KO mice each injected with AAV-GFAP-GFP or AAV-GFAP-Clu. **d** Cumulative distribution of amplitude (left) and average mEPSC amplitude of KO neurons infected with GFP or Clu viruses (*n* = 19 each). **e** Representative dendritic spines of Thy1-GFP-labeled dendrites of area CA1 of hippocampal pyramidal neurons of four-month-old *Clu* KO injected with AAV-GFAP-GFP (KO;GFP) or AAV-GFAP-Clu (KO;Clu). IMARIS softward was used to reconstruct the 3D rendering of the spines on dendrites based on confocal images of GFP-positive neurons and to classify the four spine types (mushroom, stubby, long-thin, and filopodia). Scale bar, 2 μm. **f** Total spine density in KO;GFP and KO;Clu mouse brains. **g** Density of each of the four spine types in secondary apical dendrites. Dendritic segments were derived from 5 random neurons per mouse/ 4 animals for each group. Number of animals tested for mEPSCs: *N* = 3 per group. All data are presented as mean ± SEM. *p < 0.05; ∗∗*p* < 0.01; ∗∗∗*p* < 0.001 (Student’s t test)

We next injected AAV-GFAP-Clu to *Clu* KO mice to evaluate whether astrocytic Clu expression can rescue the synaptic deficit in the mutant mice. Western blotting detected similar patterns of full-length and cleaved Clu in *Clu* KO brain infected with AAV-GFAP-Clu compared to the wild-type mice (Fig. 6a). Measurement of excitatory synaptic strength by recording mEPSCs showed that, compared to GFP infected controls, astroglial Clu expression in *Clu* KO mice significantly increased mEPSC frequency, but not amplitude, in CA1 pyramidal neurons (Fig. 6b-d). Additionally, we injected AAV-GFAP-Clu to *Clu* KO mice crossed with Thy1-GFP mice and analyzed the number of dendritic spines in Thy1-GFP-labeled CA 1 pyramidal neurons (Fig. 6e). We found that the densities of total spines and spine sub-types in apical dendrites were both increased in AAV-GFAP-Clu injected KO mice compared to GFP injected controls (Fig. 6f & g). Indeed, the total spine density in AAV-GFAP-Clu injected *Clu* KO mice was comparable with that of the WT mice (compare Fig. 6f KO;Clu with Fig. 3b WT). Thus, astrocytic expression of Clu is sufficient to rescue the synaptic deficits present in global *Clu* knockout mice.

### Astrocytic Clu overexpression ameliorates AD neuropathology and rescues synaptic deficits in 5XFAD mice

**Fig. 7:**
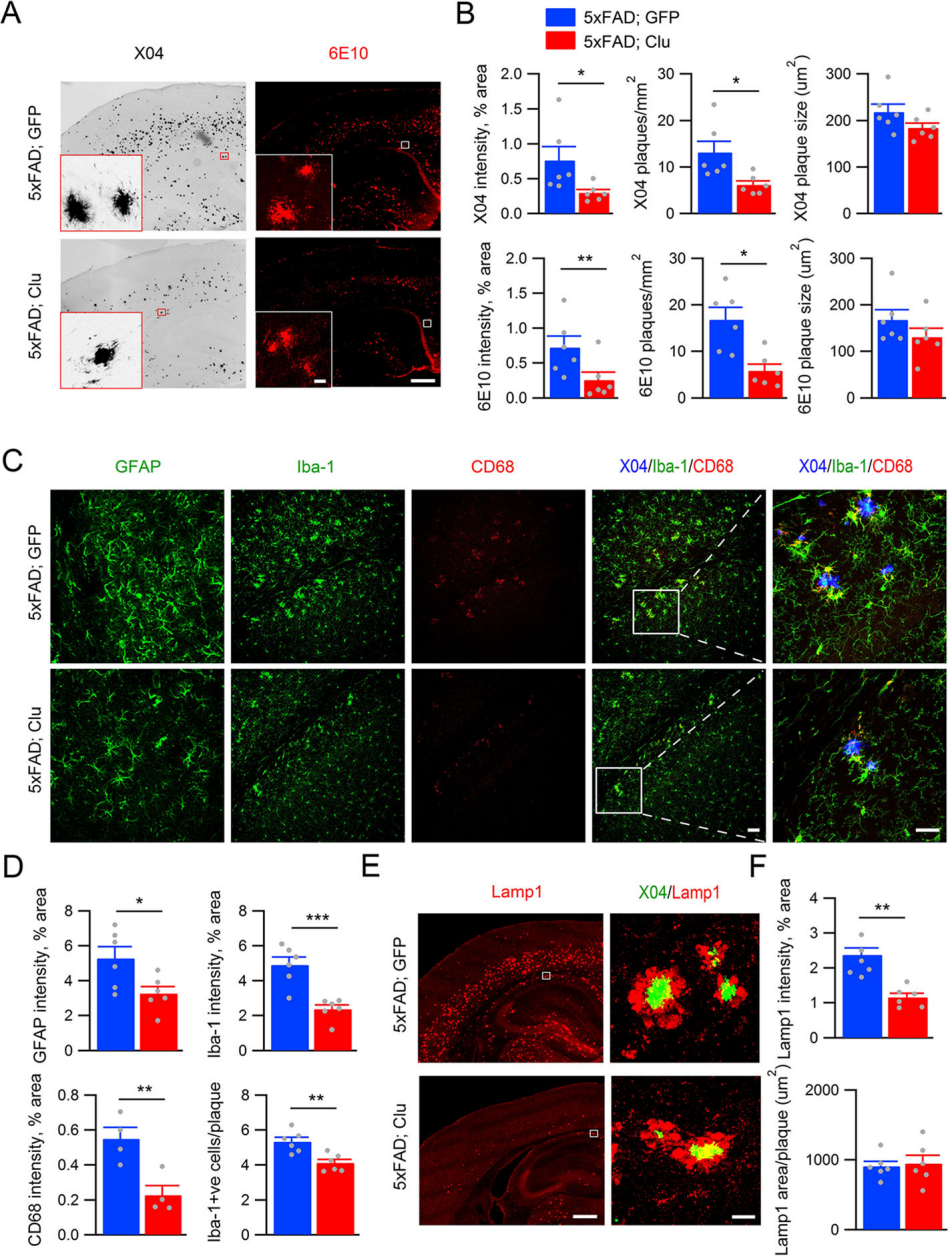
Astrocytic Clu overexpression ameliorates amyloid pathology and glial reactivity in 5xFAD mice. **a** Representative images of methoxy-X04 and 6E10 staining of 5xFAD mouse brains injected with GFP or Clu. Scale bar, 500 μm (insert 50 μm). **b** Quantification of X04 or 6E10 intensities, plaque numbers and plaque size. **c** Representative immunofluorescence images using anti-GFAP, -Iba-1 and -CD68 antibodies. Scale bar, 50 μm. **d** Quantification of immunointensitivites. **e** Representative anti-Lamp1 immunofluorescence images. **f** Quantification of Lamp1 immunoreactivities. *n* = 6/group. Scale bar, 500 μm, second from right; 50 μm, right for both (**c**) & (**e**). All data are presented as mean ± SEM. *p < 0.05; ∗∗*p* < 0.01; ∗∗∗*p* < 0.001 (Student’s t test)

Having established a physiological function of astrocytic Clu in promoting synaptic structure and excitatory synaptic transmission, we next investigated how Clu upregulation impacts synaptic properties and neuropathology relevant to AD. We chose the 5XFAD mice that exhibit aggressive amyloid deposition and synaptic deficits detectable at 4 months of age [31]. Consistent with previous reports [36], we observed an increased expression of Clu in 5XFAD animals (Fig. S2). Besides astrocytes, Clu immunoreactivity can be found associated with amyloid plaques as well as in vessels of cerebral amyloid angiopathy (Fig. S3). Prominent astroglial Clu overexpression with characteristic diffuse patterns can be detected in both the cortex and hippocampus of 5XFAD mice injected with AAV-GFAP-Clu (Fig. S4). We first evaluated the effect of Clu overexpression on Aβ pathology. Only female mice were used to reduce gender-based variabilities. Staining of brain sections of 4 month-old 5XFAD mice injected with GFP or Clu viruses with methoxy-X04, which recognizes β-sheet conformation, and 6E10, which identifies diffused and aggregated Aβ, both showed that Clu overexpression markedly reduced the total coverage and the number of amyloid deposits without affecting the size of the plaques in the cortex (Fig. 7a & b) and hippocampus (Fig. S5) of 5XFAD mice. This is associated with reduced glial relativities visualized by Iba1, GFAP and CD68 immunoreactivities (Fig. 7c & d), as well as the dystrophic neurites detected by Lamp1 immunostaining (Fig. 7e & f).

**Fig. 8:**
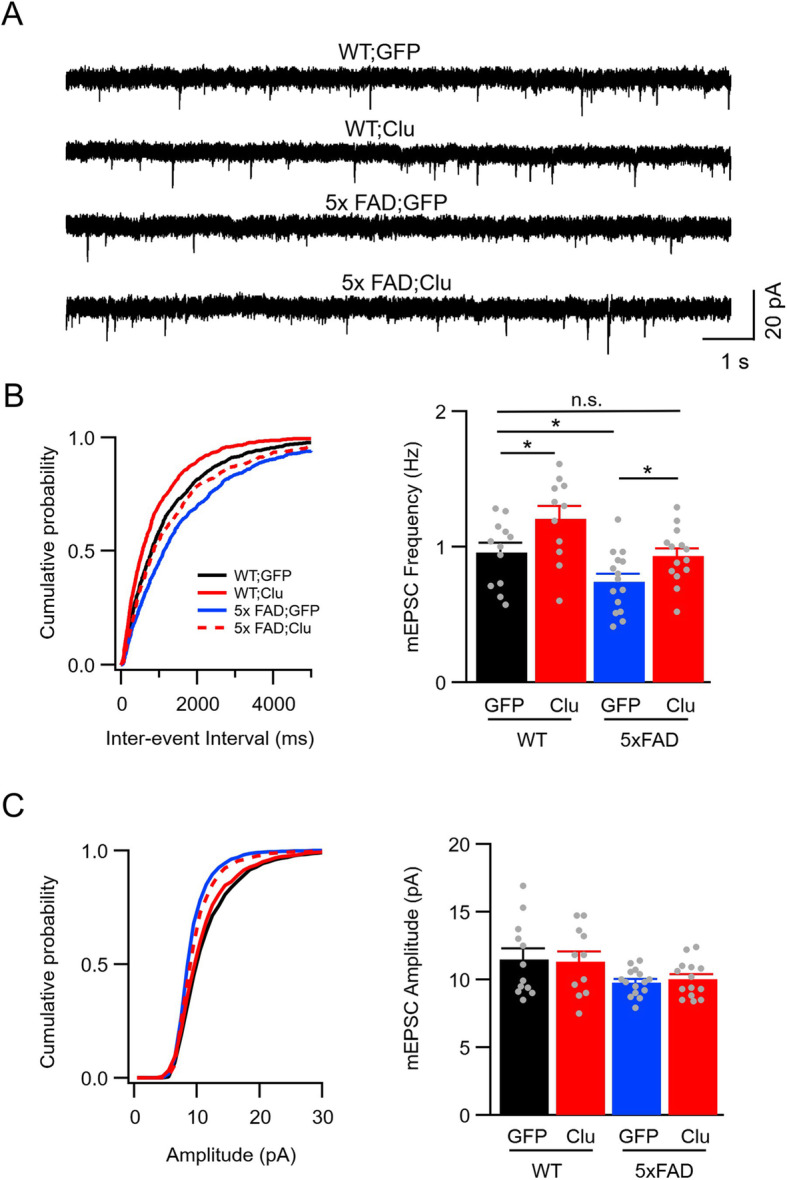
Astrocytic Clu expression rescues synaptic dysfunction in 5xFAD mice. **a** mEPSC sample traces from hippocampal CA1 pyramidal neurons of WT and 5xFAD mice injected with AAV-GFAP-GFP or AAV-GFAP-Clu (scale bar, 20 pA/1 s). **b** Cumulative distribution of inter-event interval from all mEPSCs events (left) and average mEPSC frequency (right) (*n* = 12 WT; GFP; *n* = 15 WT; Clu; *n* = 11 5xFAD; GFP; *n* = 14 5xFAD; Clu). **c** Cumulative distribution of amplitude (left) and average mEPSC amplitude (right) (*n* = 12 WT; GFP; *n* = 15 WT; Clu; *n* = 11 5xFAD; GFP; *n* = 14 5xFAD; Clu). Number of animals tested: *N* = 3 per group. Data presented are mean ± SEM. *p < 0.05; n.s. not significant (*p* > 0.05) (Two-way ANOVA)

Lastly, we investigated whether astrocytic Clu overexpression could rescue the synaptic impairment previously reported in 5XFAD mice [37]. We recorded mEPSCs of CA1 pyramidal neurons of brain slices of 4 month-old WT and 5X FAD mice injected with AAV-GFAP-GFP or AAV-GFAP-Clu (Fig. 8). We observed significant decreases in mEPSC frequency in neurons from 5X FAD brains compared with WT controls (Fig. 8b). In contrast, mEPSC amplitude was comparable among all groups (Fig. 8c). Strikingly, Clu overexpression in 5X FAD mice restored the mEPSC frequency to WT levels (Fig. 8b, WT GFP vs. 5xFAD Clu, not significant), demonstrating a complete rescue of presynaptic dysfunction of 5X FAD mice by Clu overexpression. Given that Aβ pathology is only partially attenuated, the results suggest that the complete rescue of synaptic function by astrocytic Clu is due to its combined effects in synapse enhancement and Aβ antagonism.

## Discussion

Clusterin is a well-established genetic modifier for LOAD. Since both risk and protective alleles have been identified which may be associated with its expression levels, loss- and gain-of-function studies of Clu may both reveal important insights into its genetic underpinning. Using *Clu* deficient mice and by AAV-mediated Clu expression in astrocytes, we reveal here that Clu secreted from astrocyte is co-localized to the presynaptic site of excitatory neurons where it modulates excitatory synaptic transmission and spine density. Astroglial Clu expression is sufficient to rescue the impaired synaptic structure and function in *Clu* knockout mice and led to the attenuation of amyloid pathology and synaptic deficit in 5XFAD mice.

Astrocytes are increasingly recognized to play critical roles in synapse formation and synaptic function through their physical proximity with neurons and by secreting synaptotrophic factors [38]. For example, astrocytes secrete thrombospondins to promote synaptogenesis [39, 40], hevin and SPARC to control synapse formation [41], and glypican 4 and 6 to induce functional synapses [42]. In addition to these synaptogenic factors, several other molecules including transforming growth factor β [43], D-Serine [44] and cholesterol associated with apolipoprotein E (ApoE) [45] have been identified as astrocyte derived factors that regulate specific steps of synapse formation via interaction with neuronal receptors. We present evidence that Clu acts as an astrocyte secreted factor facilitating synapse formation and excitatory synaptic transmission. Specifically, endogenous Clu is predominantly expressed in astrocytes but is also localized close to the synaptic sites of excitatory neurons. Expression of Clu in sparse astrocytes of *Clu* knockout mice revealed the presence of Clu in both astrocyte cell body and fine distal processes in close proximity to vGlut1-marked synaptic puncta where it may be released. Loss of Clu results in impaired glutamatergic synaptic transmission and reduced dendritic spine density, and these phenotypes can be rescued by astrocytic expression of Clu. We show that Clu promotes excitatory but not inhibitory synaptic transmission. This is likely attributed by its selective association with glutamatergic presynaptic marker vGlut1. The reason for this specificity and whether this is mediated by a direct interaction between Clu and vGlut1 or through other interacting proteins are not clear. In this regard, several putative Clu receptors have been reported including low-density lipoprotein receptor related protein 2 (LRP2) [46, 47], LRP8 [48], very low-density lipoprotein receptor [48], Plexin A4 [49], and triggering receptor expressed on myeloid cells 2 (TREM2) [50]. Whether these receptors are involved in mediating the synaptic localization and function of Clu remain to be investigated. Interestingly, a recent report demonstrated a role of TREM2 signaling in synapse sculpting [51]. Using live-cell imaging, a separate study revealed that microglia frequently engulf presynaptic, but not postsynaptic, site whereby participating synaptic remodeling and maintenance [52]. Thus it is tempting to speculate that the presynaptic function of Clu may involve microglia and Clu-TREM2 interaction.

We report here that astrocytic Clu overexpression leads to a drastic reduction of parenchymal Aβ pathology and associated glial reactivity and dystrophic neurite accumulation in 5XFAD model. This may be mediated by the following mechanisms, all involving Clu-Aβ interactions. Firstly, as an extracellular chaperone, Clu has been shown to bind to Aβ and affect its nucleation and aggregation [25, 53]. Thus, astrocyte secreted Clu may block Aβ deposition through this chaperone function. Secondly, Clu may promote Aβ clearance through microglia uptake. Supporting this idea, lipidated Clu has been shown to complex with Aβ and mediate Aβ clearance through binding to microglia TREM2 [50, 54]. Additionally, a recent report demonstrates that Clu facilitates the degradation of its misfolded protein clients including Aβ via heparan sulfate receptor-mediated endocytosis [55]. Lastly, Clu has been well-documented to complex with Aβ and regulate Aβ clearance through the perivascular drainage system at the blood brain barrier and/or the blood-cerebrospinal fluid barrier via LRP2 [46, 47, 56]. Thus, astrocytic Clu overexpression could drive this process and, by which means, remove parenchymal Aβ.

Studies of *Clu* knockout mice documented increased CAA in APP/PS1 transgenic mice, supporting a functional role of Clu in Aβ clearance through the vascular system [27]. However, this is associated with reduced parenchymal Aβ plaques, a finding that is not consistent with the anti-amyloidogenic properties of Clu we observed [26, 27]. Although the precise reason for the discrepancy is not clear, it may be attributed by the dynamic regulation of Clu in both Aβ fibrillogenesis and clearance, making it difficult to directly compare and contrast the loss- and gain-of-function studies. In addition, the Aβ phenotypes presented in *Clu* null mice may be confounded by genetic redundancies since work done by DeMattos et al. clearly demonstrated an additive effect of ApoE and Clu in Aβ metabolism [57].

Our results show that astroglial Clu expression completely rescued the presynaptic deficit in 5XFAD mice although the Aβ pathology is only partially reduced, suggesting the Clu has a direct effect on Aβ-induced synaptic toxicity independent of amyloid pathology. It is known that neuronal activity evokes the release of Aβ at the synapse, leading to increased levels of extracellular Aβ at or near synapses [58]. Abnormal assemblies of Aβ suppress excitatory transmitter release [59]. It is possible that astrocyte-secreted and synaptically targeted Clu could improve synaptic transmission by mediating the clearance of this Aβ pool and/or by interacting with vGlut1 and enhancing basal synaptic transmission.

Elevated levels of CLU have been observed in the brain and cerebrospinal fluid of AD patients [17–20]. Our findings that Clu overexpression suppresses Aβ pathology and restores synaptic function support the idea that this increase serves as an adaptive mechanism to respond to ongoing neuronal insults and Aβ stress, especially at early stages of AD pathogenesis. However, overwhelming accumulation of Aβ may lead to its sequestration and reduced capacity for synaptic maintenance and Aβ clearance. This assessment is consistent with genetic evidence that the protective allele of *CLU* may be associated with increased expression and vice versa [5–8].

## Conclusions

Our study provides evidence for an essential role of Clu secreted from astrocyte in modulating excitatory synaptic function and amyloid pathogenesis. Elevating CLU levels in the brain may afford protection against AD by enhancing synaptic transmission and through Aβ reduction.

## Supplementary Information

**Additional file 1: Table S1**. The top 20 enriched Reactome pathways differentially regulated by Clu. **Figure S1. **Widespread astroglial expression by AAV-GFAP-GFP. **Figure S2**. Increased Clu expression in the astrocyte of 5XFAD mice. **Figure S3**. Association of Clu with amyloid plaques and cerebral amyloid angiopathy. **Figure S4**. Expression of AAV-GFAP-Clu in 5XFAD mouse brains. **Figure S5**. Astrocytic Clu reduces amyloid load in the cortex of 5XFAD mice

## Data Availability

The datasets used and/or analyzed during the current study are available from the corresponding author on reasonable request.

## Abbreviations

Aβ: Amyloid beta
ACSF: Artificial cerebrospinal fluid
AD: Alzheimer’s disease
ApoE: Apolipoprotein E
CAA: Cerebral amyloid angiopathy
Clu: Clusterin
CSF: Cerebrospinal fluid
CM: Conditioned medium
fEPSPs: field excitatory postsynaptic potentials
GFAP: Glial fibrillary acidic protein
GO: Gene ontology
KO: Knockout
LRP: lipoprotein receptor related protein
LOAD: Late-onset AD
mEPSCs: miniature excitatory postsynaptic currents
mIPSCs: miniature inhibitory postsynaptic currents
PANTHER: Protein Analysis Through Evolutionary Relationships
PBS: Phosphate-buffered saline
PDL: Poly-D-lysine
PFA: Paraformaldehyde
PPR: Paired-pulse ratio
TREM2: Triggering receptor expressed on myeloid cells 2
vGlut1: vesicular glutamate transporter 1
WT: Wild-type
