# Supplementary material for: Clusterin secreted from astrocyte promotes excitatory synaptic transmission and ameliorates Alzheimer’s disease neuropathology
Source: Mol Neurodegener. 2021 Jan 31;16:5. doi: 10.1186/s13024-021-00426-7 (PMC7849119; doi:10.1186/s13024-021-00426-7)
Supplement: Supplementary file 1 — Additional file 1: Table S1. The top 20 enriched Reactome pathways differentially regulated by Clu. Figure S1. Widespread astroglial expression by AAV-GFAP-GFP. Figure S2. Increased Clu expression in the astrocyte of 5XFAD mice. Figure S3. Association of Clu with amyloid plaques and cerebral amyloid angiopathy. Figure S4. Expression of AAV-GFAP-Clu in 5XFAD mouse brains. Figure S5. Astrocytic Clu reduces amyloid load in the cortex of 5XFAD mice [file 13024_2021_426_MOESM1_ESM.pdf]

Supplementary Materials

Table S1. The top 20 enriched Reactome pathways differentially regulated by Clu.

Figure S1. Widespread astroglial expression by AAV-GFAP-GFP.

Figure S2. Increased Clu expression in the astrocyte of 5XFAD mice.

Figure S3. Association of Clu with amyloid plaques and cerebral amyloid angiopathy.

Figure S4. Expression of AAV-GFAP-Clu in 5XFAD mouse brains.

Figure S5. Astrocytic Clu reduces amyloid load in the cortex of 5XFAD mice.

Table S1. The top 20 enriched Reactome pathways differentially regulated by Clu.

No	Pathway name	No. of genes	P-value (FDR)
1	Neuronal System	18	1.77E-07
2	Transmission across Chemical Synapses	15	3.68E-07
3	Neurotransmitter receptors and postsynaptic signal transmission	11	1.61E-05
4	Axon guidance	16	2.21E-05
5	Protein-protein interactions at synapses	7	1.31E-04
6	Activation of NMDA receptors and postsynaptic events	7	3.68E-04
7	L1CAM interactions	7	7.39E-04
8	Synaptic adhesion-like molecules	4	7.39E-04
9	Post NMDA receptor activation events	6	0.001184949
10	Unblocking of NMDA receptors, glutamate binding and activation	4	0.001267048
11	Neurexins and neuroligins	5	0.001363694
12	Opioid Signaling	6	0.002084152
13	Activation of Ca-permeable Kainate Receptor	3	0.002223415
14	Trafficking of AMPA receptors	4	0.002223415
15	Ionotropic activity of kainate receptors	3	0.002223415
16	truncated APC mutants destabilize the destruction complex	3	0.002223415
17	Truncations of AMER1 destabilize the destruction complex	3	0.002223415
18	AXIN missense mutants destabilize the destruction complex	3	0.002223415
19	APC truncation mutants have impaired AXIN binding	3	0.002223415
20	Glutamate binding, activation of AMPA receptors and synaptic plasticity	4	0.002223415

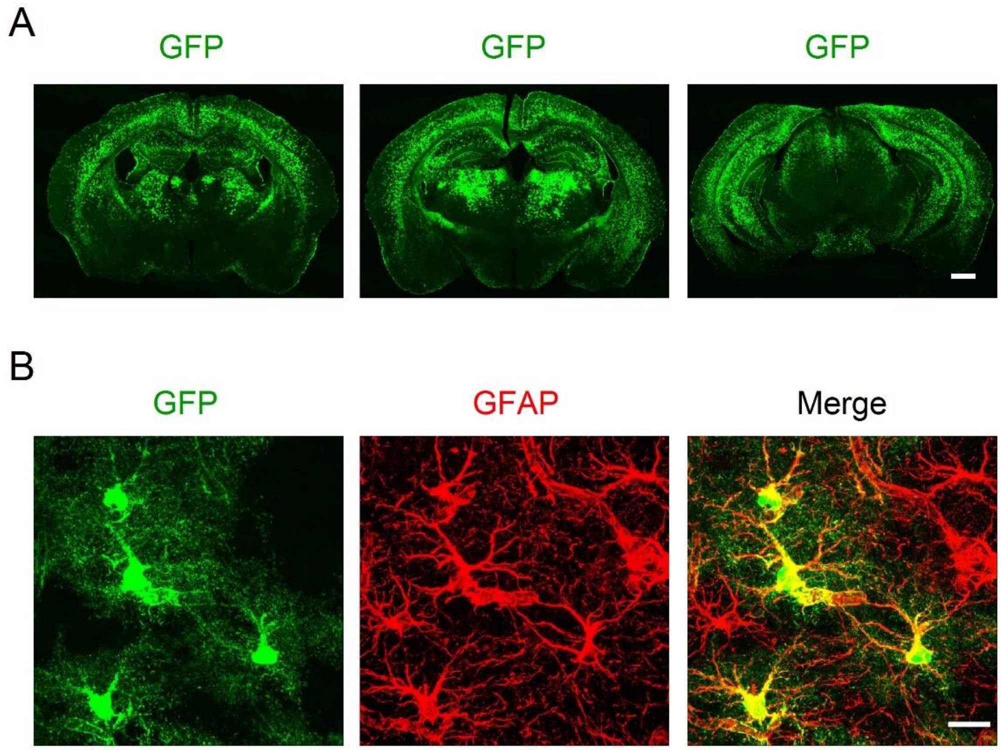

Figure S1. Widespread astroglial expression by AAV-GFAP-GFP. A. Representative fluorescence images of successive coronal sections of a wild-type mouse brain intraventricularly injected with AAV-GFAP-GFP at P3. Scale bar, 1 mm. **B.** High resolution confocal immunofluorescence images of GFP (green) and GFAP (red) in CA1 of hippocampus showing GFP expression in GFAP-positive astrocytes. Scale bar, 30 µm.

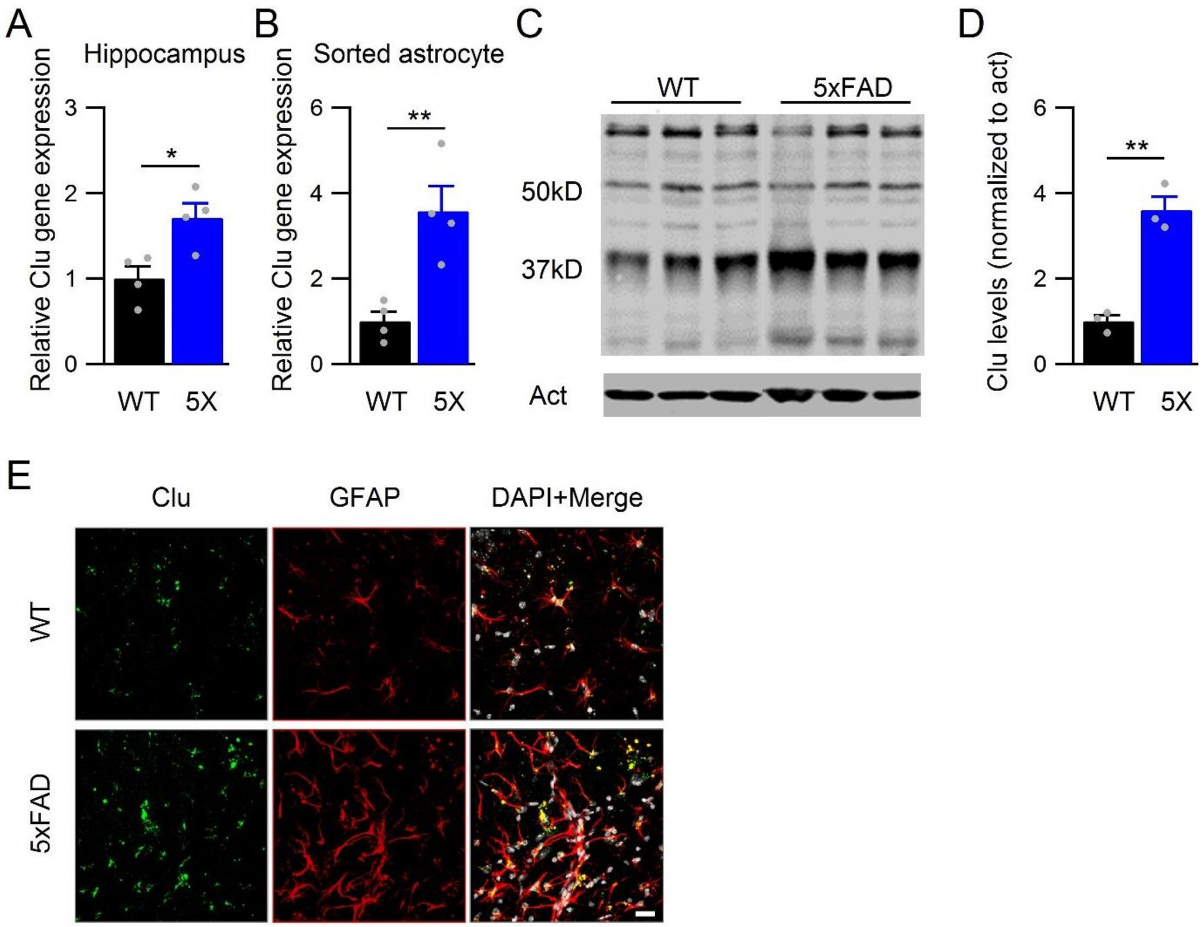

Figure S2. Increased Clu expression in the astrocyte of 5XFAD mice. A&B. Relative *Clu* mRNA levels by qPCR in bulk hippocampus [A] and sorted astrocytes [B]. n=4/group. **C.** Western blotting of Clu expression in hippocampal tissues of wild-type and 5XFAD mice. **D.** Quantification of Clu expression (37 kD) normalized to Actin. n= 3/group. **E.** Representative confocal images of Clu (green) and GFAP (red) immunostaining of hippocampal CA1 area of WT and 5XFAD mice. Scale bar, 40 µm. All data are presented as mean ± SEM. *p < 0.05; **p < 0.01 (Student's t test).

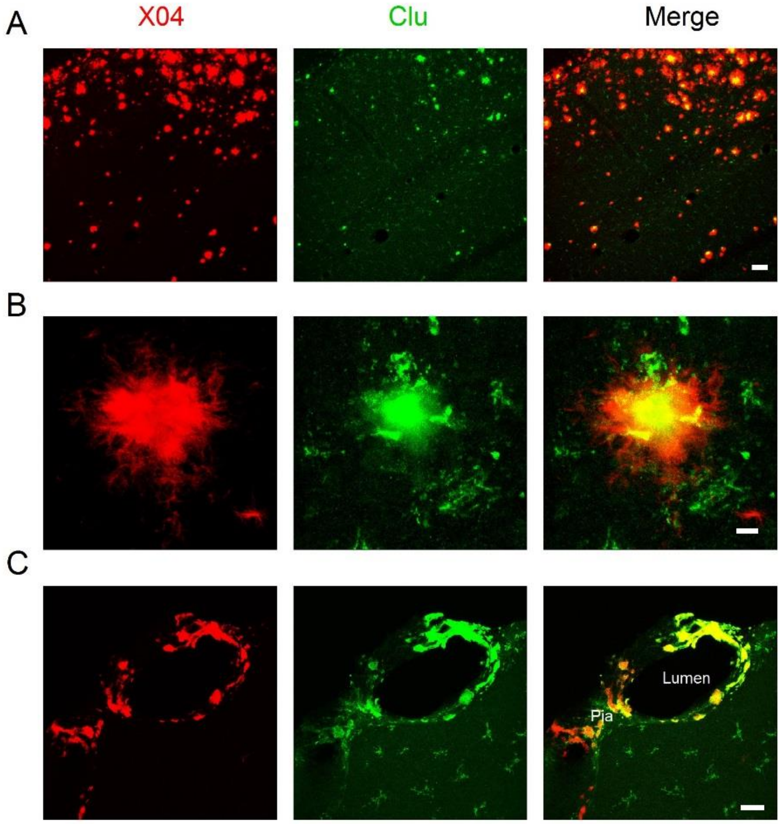

Figure S3. Association of Clu with amyloid plaques and cerebral amyloid angiopathy. Representative confocal images of X04 (red) and Clu (green) immunostaining in the hippocampus of 5xFAD mice [**A**], a single plaque zoom in [**B**] and a surface vessel [**C**]. Scale bar in A, 40 µm; in B, 5 µm; in C, 20 µm.

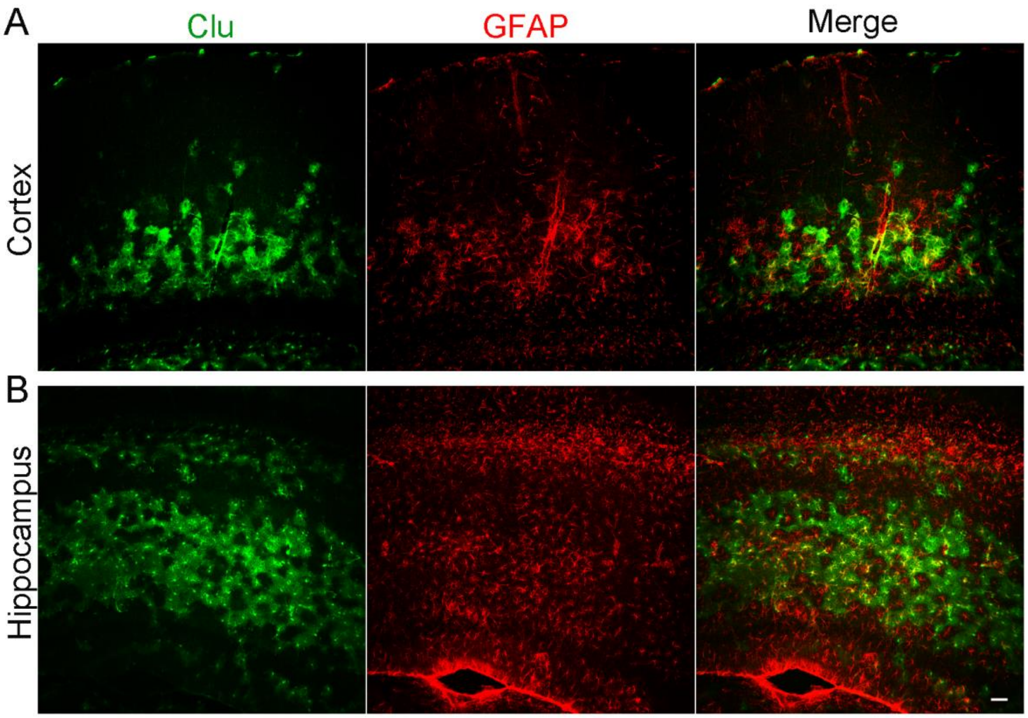

Figure S4. Expression of AAV-GFAP-Clu in GFAP-positive astrocytes of 5XFAD mouse brains. Representative confocal images of Clu (green) and GFAP (red) immunostaining in the cortex [**A**] and hippocampus [**B**] of 5XFAD mice injected with AAV-GFAP-Clu at P3. Images are taken from a 4 month-old mouse brain. Scale bar, 50 µm.

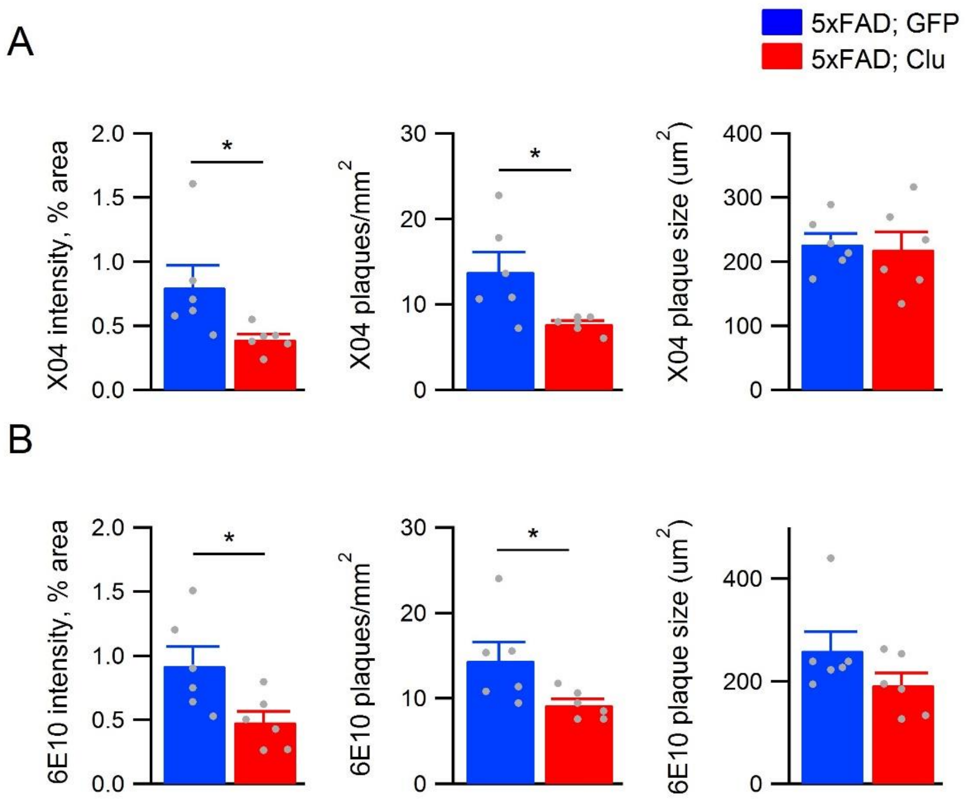

Figure S5. Astrocytic Clu reduces amyloid load in the cortex of 5XFAD mice. Quantification of X04 [**A**] or 6E10 [**B**] positive areas, plaque numbers and plaque size in 5xFAD mouse brains injected with AAV-GFAP-GFP or AAV-GFAP-Clu. n=6/group. All data are presented as mean ± SEM. *$p < 0.05$ (Student's t test).
